# Peak flow measurements in patients with severe aortic stenosis: a prospective comparative study between cardiovascular magnetic resonance 2D and 4D flow and transthoracic echocardiography

**DOI:** 10.1186/s12968-021-00825-1

**Published:** 2021-11-15

**Authors:** Reetta Hälvä, Satu M. Vaara, Juha I. Peltonen, Touko T. Kaasalainen, Miia Holmström, Jyri Lommi, Satu Suihko, Helena Rajala, Minna Kylmälä, Sari Kivistö, Suvi Syväranta

**Affiliations:** 1 Radiology, HUS Diagnostic Center, University of Helsinki and Helsinki University Hospital, Helsinki, Finland; 2 Heart and Lung Center, University of Helsinki and Helsinki University Hospital, Helsinki, Finland

**Keywords:** Aortic valve stenosis, 4D flow, 2D flow, Cardiovascular magnetic resonance, Valvular heart disease

## Abstract

**Background:**

Aortic valve stenosis (AS) is the most prevalent valvular disease in the developed countries. Four-dimensional (4D) flow cardiovascular magnetic resonance (CMR) is an emerging imaging technique, which has been suggested to improve the evaluation of AS severity compared to two-dimensional (2D) flow and transthoracic echocardiography (TTE). We investigated the reliability of CMR 2D flow and 4D flow techniques in measuring aortic transvalvular peak systolic flow in patients with severe AS.

**Methods:**

We prospectively recruited 90 patients referred for aortic valve replacement due to severe AS (73.3 ± 11.3 years, aortic valve area 0.7 ± 0.1 cm^2^, and 54/36 tricuspid/bicuspid), and 10 non-valvular disease controls. All the patients underwent echocardiography and 2D flow and 4D flow CMR. Peak flow velocity measurements were compared using Wilcoxon signed rank sum test and Bland–Altman analysis.

**Results:**

4D flow underestimated peak flow velocity in the AS group when compared with TTE (bias − 1.1 m/s, limits of agreement ± 1.4 m/s) and 2D flow (bias − 1.2 m/s, limits of agreement ± 1.6 m/s). The differences between values obtained by TTE (median 4.3 m/s, range 2.7–6.1 m/s) and 2D flow (median 4.5 m/s, range 2.9–6.5 m/s) compared to 4D flow (median 3.1 m/s, range 1.7–5.1 m/s) were significant (p < 0.001). The difference between 2D flow and TTE were insignificant (bias 0.07 m/s, limits of agreement ± 1.5 m/s). In non-valvular disease controls, peak flow velocity was measured higher by 4D flow than 2D flow (1.4 m/s, 1.1–1.7 m/s and 1.3 m/s, 1.1–1.5 m/s, respectively; bias 0.2 m/s, limits of agreement ± 0.16 m/s).

**Conclusions:**

CMR 4D flow significantly underestimates systolic peak flow velocity in patients with severe AS. 2D flow, in turn, estimated the AS velocity accurately, with measured peak flow velocities comparable to TTE.

**Supplementary Information:**

The online version contains supplementary material available at 10.1186/s12968-021-00825-1.

## Introduction

Aortic valve stenosis (AS) is the most prevalent valvular disease among adults [1, 2], and severe, symptomatic AS without treatment has poor prognosis [3]. The accurate evaluation of disease severity is essential in determining the need for valve replacement/intervention [4, 5]. The diagnostic gold standard is transthoracic echocardiography (TTE), including the assessment of mean transvalvular pressure gradient and peak aortic jet velocity. Definition of the severity of the valvular obstruction may be confounded by poor visibility on TTE and some patients also present with discordant ultrasound findings supporting the need for alternative imaging methods.

Transvalvular flow conditions can also be evaluated by cardiovascular magnetic resonance (CMR). In fact, the emerging CMR four-dimensional (4D) phase-contrast flow technique has already shown potential in the field of valvular disease, for instance in bicuspid valve-related aortic disease, and also in AS [6–8]. 4D flow CMR has been expected to overcome some of the issues related to standard two-dimensional (2D) flow CMR, which has been shown to underestimate the turbulent and angulated flow jets caused by the narrowed orifice in AS [9, 10], the most accurate velocity values being obtained close to the valve level [11, 12]. Indeed, promising results on 4D flow being superior to 2D technique in measuring peak flow velocity in mild to moderate stenosis have emerged [13]. Its accuracy in severely stenotic valves has, however, remained unclear.

In this study, we compare CMR 4D flow with 2D flow and TTE in patients with severe AS. We aimed to investigate whether the CMR flow measurement techniques are reliable in measuring peak systolic flow velocity in a routine workflow in this patient group, and therefore a feasible diagnostic alternative in severe stenosis.

## Methods

### Study population

In this prospective cohort study, we recruited patients referred for aortic valve replacement either by open heart surgery or transcatheter aortic valve implantation (TAVI) due to suspected symptomatic, severe AS (peak transvalvular flow velocity ≥ 4.0 m/s, and mean pressure gradient ≥ 40 mmHg or aortic valve area ≤ 1.0 cm^2^). Patients with other significant (more than mild or moderate) valvular disease were excluded.

A total of 150 patients were evaluated by TTE, and 95 patients were referred for CMR. We recruited consecutive AS patients who gave their informed consent and were able to undergo CMR. Therefore, patients with incompatible implants, pacemakers, severe obesity, decompensation or other instability, and those not able to lie flat on the CMR table for 45 min, were not referred for CMR. Out of the referred 95 patients, one patient refused CMR due to claustrophobia, and one patient was excluded due to insufficient image quality. In addition, three patients initially referred for CMR were found to have only moderate stenosis. The remaining 90 patients were all included in the analyses. For detailed patient characteristics, see Table 1. We also recruited 10 subjects without valvular disease.

**Table 1:** Baseline characteristics of the study population

	N	Mean ± SD or n of patients	Range
Sex, male/female	90	51/39	
Age (years)	90	73.3 ± 11.3	46.0–93.8
Height (cm)	84	170.2 ± 10.5	142–188
Weight (kg)	84	80.9 ± 16.2	38–118
BMI (kg/m^2^)	84	27.8 ± 4.4	18.9–37.7
Heart rate (bpm)	59	67 ± 13	45–120
eGFR	89	75 ± 18	21–107
Hypertension	90	66 (73.3%)	
Dyslipidemia	90	73 (81.1%)	
Diabetes	90	20 (22.2%)	
Smoker	89	31 (34.8%)	
Morbus cordis coronaries	87	36 (41.4%)	
NYHA class	74		
I		3 (4.1%)	
II		47 (63.5%)	
III		22 (29.7%)	
IV		2 (2.7%)	
Valve morphology	90		
Bicuspid		36 (40.0%)	
Tricuspid		54 (60.0%)	
TTE values			
LVEF (%)	90	60.0 ± 9.2	35.0–78.0
LVEDD (mm)	90	49.1 ± 6.8	31.0–73.0
LVESD (mm)	70	32.8 ± 7.5	20.0–60.0
AVA (cm^2^)	89	0.7 ± 0.1	0.4–1.0
Mean gradient (mmHg)	89	46 ± 14	15.0–90.0
Vmax (m/s)	90	4.3 ± 0.6	2.7–6.1
LFLG-AS	90	4 (4%)	

*BMI* body mass index, *eGFR* estimated glomerular filtration rate, *NYHA* New York Heart Association, *TTE* transthoracic echocardiogram, *LVEF* left ventricular ejection fraction, *LVEDD* left ventricular end-diastolic diameter, *LVESD* left ventricular end-systolic diameter, *AVA* aortic valve area, *Vmax* maximum velocity, *LFLG-AS* low flow–low gradient aortic stenosis

### Echocardiography

TTE studies were performed and evaluated by experienced cardiologists according to international diagnostic guidelines [14, 15] using Vivid e9 (General Electric Healthcare, Chicago, Illinois, USA) or EPIC (Philips Healthcare, Best, the Netherlands) echocardiographs. In particular, left ventricular ejection fraction (LVEF) was determined from the apical four-chamber (4Ch) view using Simpson’s formula and dimensions of the heart chambers were analyzed using the 2D imaging planes. Grading of AS was performed according to the European Society of Cardiology and American Heart Association guidelines using peak aortic jet velocity and mean pressure gradient [16]. The aortic valve area (AVA) was calculated with the continuity equation.

### CMR imaging

**Fig. 1:**
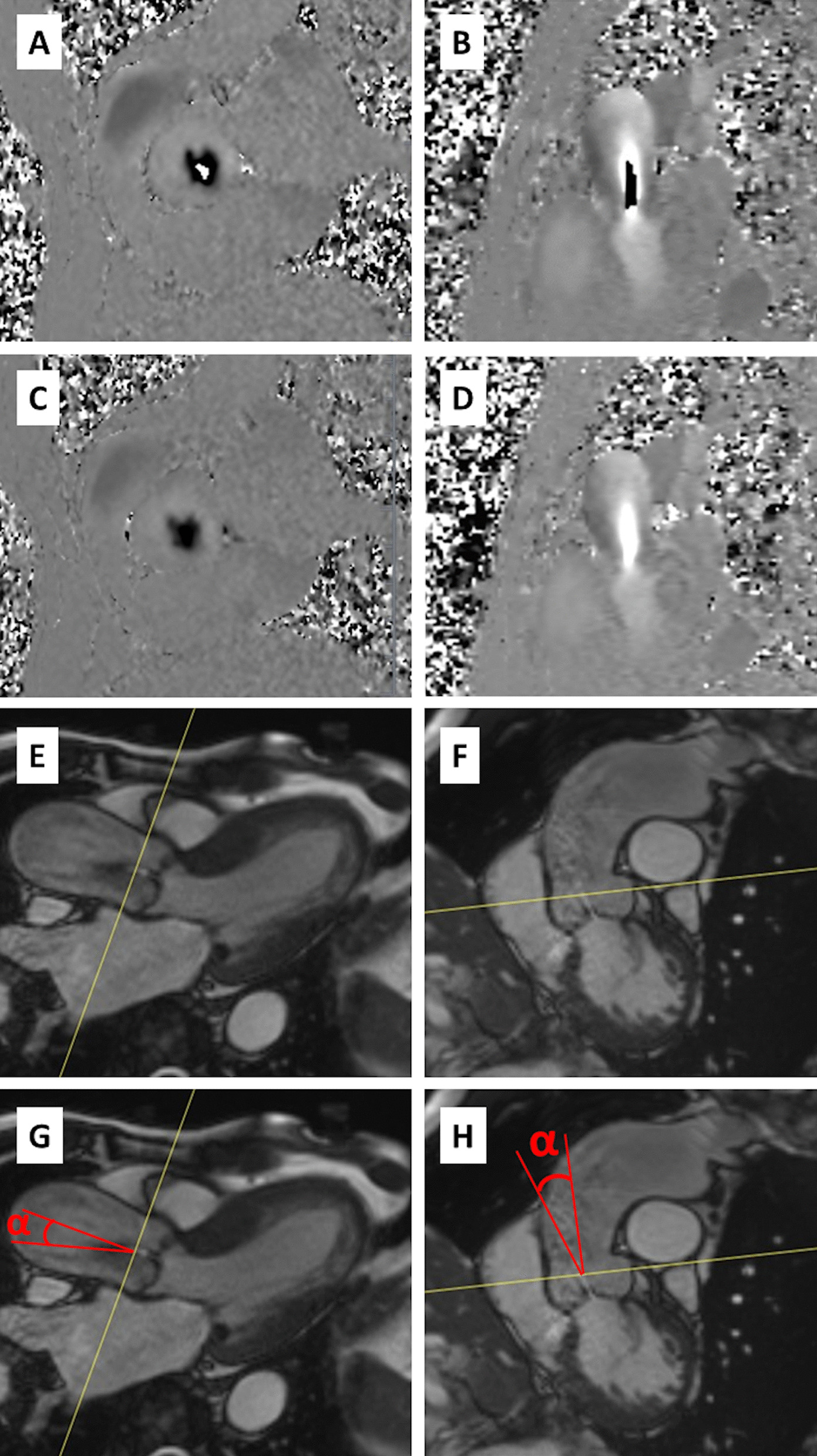
2D flow analyses. **A**–**D** Inplane left ventricular outflow tract (LVOT) and throughplane phase-contrast images with aliasing (**A** and **B**) and with corrected velocity encoding settings without aliasing (**C**, **D**). **E**–**H** 2D flow jet angle measurements from LVOT and coronal oblique planes, coronal oblique plane demonstrating the widest angle selected for the variable calculation (**H**)

CMR, including flow imaging, was performed according to international guidelines and consensus statements [17–21]. The mean interval between the TTE and CMR was 14 ± 17 days. The images were acquired using a 1.5T CMR system (MAGNETOM AvantoFit, Siemens Healthineers, Erlangen, Germany) by two experienced radiographers. We assessed the morphology of the valves visually from balanced steady-state free precession (bSSFP) cine images. Experienced cardiac radiologists (certified European Association of Cardiovascular Imaging level 3) approved all the reports and measurements. All 100 CMR studies included 2D and 4D phase-contrast imaging. The optimal maximum velocity encoding (VENC) value for 2D flow was determined using in-plane 3-chamber left ventricular outflow tract (LVOT) VENC scout images set at 0.5 m/s intervals from 3 to 5 m/s and corrected in case of visible aliasing (Fig. 1A–D). For 4D flow, VENC was set at 0.5 m/s higher than the peak flow velocity obtained at TTE or 2D flow if higher. The median VENC was 4.8 m/s (range 3.0–6.5 m/s) for 2D flow, and 4.8 m/s (range 3.5–6.5 m/s) for 4D flow. For non-valvular disease controls, the VENC was set at 2 m/s.

### CMR 2D flow

2D flow CMR was carried out in through-plane setting within a single breath-hold. Previously acquired in-plane 3-chamber (LVOT) VENC scout images were used to align the imaging plane perpendicular to the jet emerging from the valve to minimize error in peak flow acquisition. The position of the measurement plane was as close as possible to the aortic valve without risking the valve movement in the field-of-view (FOV) at the systolic phase. The used imaging parameters were: echo time (TE) 2.68 ms, repetition time (TR) 49.7 ms, echo spacing 5.0 ms, flip angle 20°, slice thickness 6 mm, and spatial resolution 1.8 × 1.8 mm^2^. Parallel imaging factor 2 with GRAPPA was used. The data were acquired with retrospective electrocardiogram (ECG) gating and reconstructed to 25 temporal phases. 2D flow measurements were analyzed using QFlow (Medis Medical Imaging Systems, Leiden, The Netherlands). Background offset correction was applied before the analysis.

Jet offset angle was determined by measuring the widest angle between flow direction and 2D flow measurement plane normal from perpendicular 3-chamber (LVOT) and coronal aortic root bSSFP cine images (Fig. 1E–H). A separate angle-corrected 2D flow variable was calculated using the formula 1/cos(α), where α is the angle between jet direction and the normal of the measurement plane.

### CMR 4D flow

In 4D flow, the velocity is encoded along all three spatial dimensions throughout the cardiac cycle. Each 4D data set contains velocity encoded volumes in three dimensions of space. The scanning was performed in free breathing with two averages. The imaging parameters of the volumes were TE 2.76 ms, TR 42.1 ms, echo spacing 5.3 ms, flip angle 15°, and acquisition voxel size 2.4 × 2.4 × 2.5 mm^3^. Parallel imaging factor 2 with GRAPPA was used. The data were acquired with prospective ECG gating and reconstructed with 42.1 ms time resolution. The mean acquisition time was 11 ± 2.2 min.

**Fig. 2:**
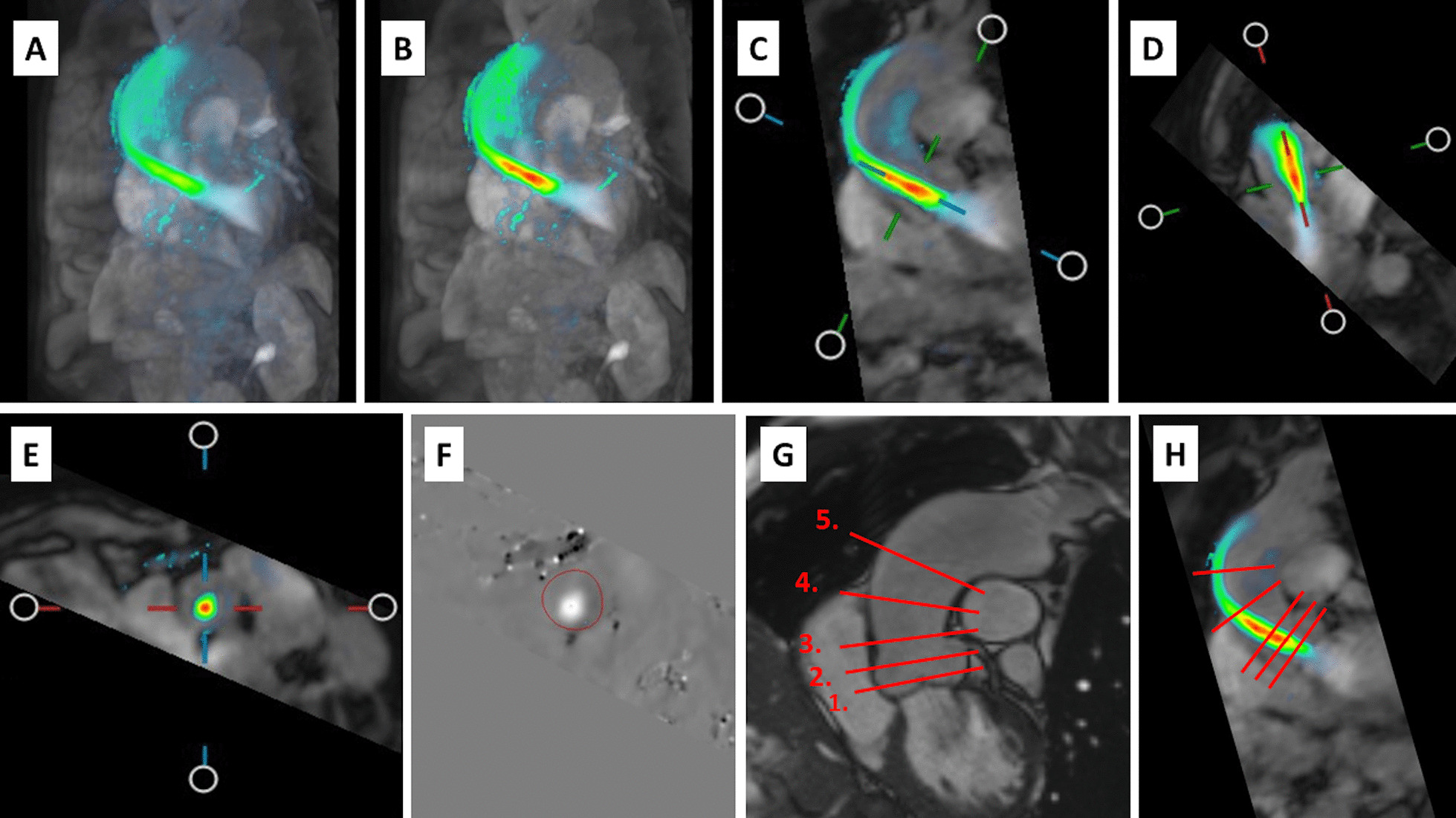
4D flow analyses. Systolic phase 4D flow image (**A**) was scaled to visualize peak flow velocity (**B**), measurement plane was selected from perpendicular planes (**C**–**E**) and analyzed (**F**) at the level of the highest flow velocity. Coronal oblique cine (**G**) and 4D flow (**H**) images demonstrating the different anatomical planes presented in Fig. 3B; valvular plane (1), the sinus of Valsalva (2), sinotubular junction (3), and the ascending aorta (4–5)

Second-order phase offset correction was applied to the imaging volume before analysis. At the beginning of the image analysis, the separate phase-contrast directions were inspected in case of velocity aliasing. No visible aliasing was detected in any of the 4D flow images and therefore antialiasing tools were not applied. The measurements were analyzed using QFlow 4D (Medis Medical Imaging Systems). Peak flow velocity measurements were made from a plane that was set perpendicular to the forward flow jet at the level and phase showing the highest flow velocity values (Fig. 2A–F). The measurements were made by two independent observers.

In order to verify the detection of maximum velocity, we analyzed a randomly-selected subpopulation of 10 patients at the same planes where the respective 2D flow measurements were made, and also from 5 anatomical planes along the aortic root and the ascending aorta (Fig. 2G, H). To further detect possible software-specific error sources, the data (89 patients and 10 controls) were also analyzed using a volumetric assessment from velocity maximum intensity projection (MIP) images as described by Rose et al. [22–24]. One patient was excluded from the 4D flow MIP analysis because of unsuccessful aortic segmentation due to anatomical variation. In addition, a subpopulation of 9 patients was also analyzed with CAAS MR 4D Flow (Pie Medical Imaging BV, AJ Maastricht, The Netherlands).

### Statistical analyses

Statistical analyses were performed using SPSS (version 27.0. Statistical Package for the Social Sciences, International Business Machines, Inc., Armonk, New York, USA). Peak flow velocity measurement techniques were compared using Bland–Altman plots. Statistical significance was set at p < 0.05 and determined using the Wilcoxon signed rank sum test. Spearman’s rank correlation coefficient was used for flow correlation analyses, and intraclass correlation coefficient was calculated to evaluate the agreement between observers.

## Results

### TTE and CMR 2D flow

**Fig. 3:**
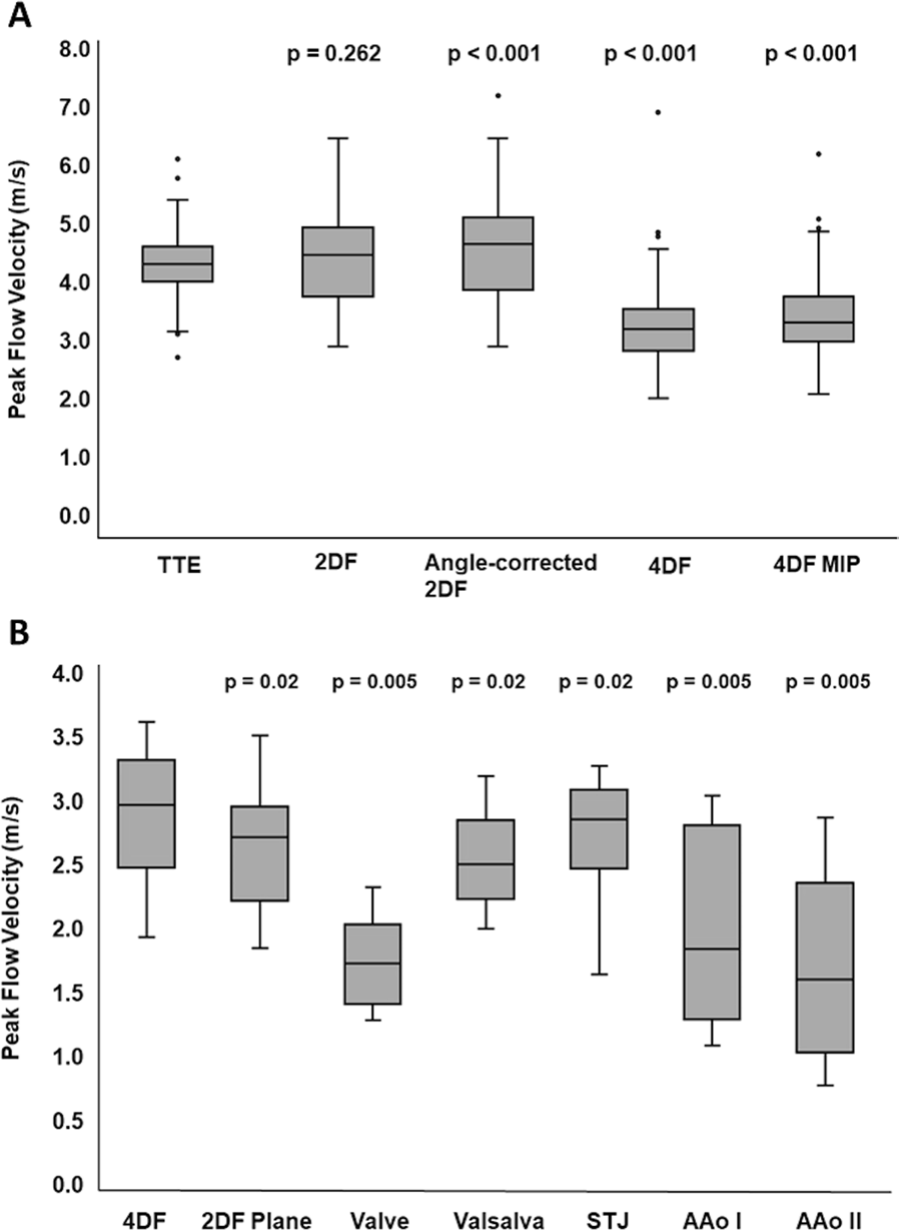
**A** Aortic peak systolic flow velocity measured from 90 patients with transthoracic echocardiography (TTE), 2D flow, 2D flow with angle correction, and 4D flow measured by the commercially available method (4D flow) and the maximum intensity projection–based method described in [23] (4D flow MIP). P-values compared to TTE. **B** Peak flow velocity measured by 4D flow from 10 patients at the level of the highest flow, 2D flow measurement plane and the five anatomical planes. P-values compared to 4D flow

**Fig. 4:**
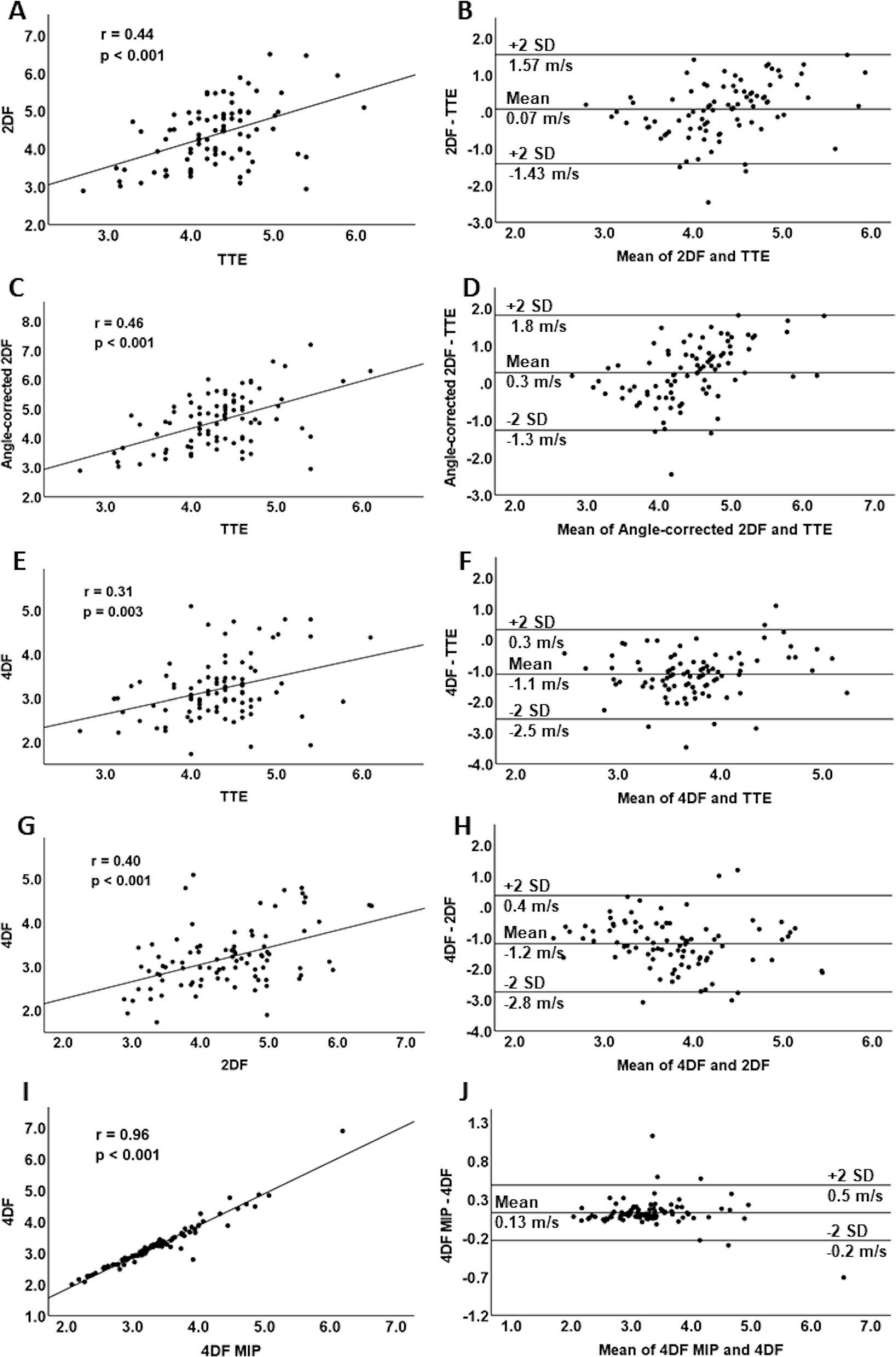
Linear regression, Spearman’s correlation and Bland–Altman analyses of the five different peak flow measurement methods. **A**, **B** 2D flow and TTE, **C**, **D** Angle-corrected 2D flow and TTE, **E**, **F** 4D flow and TTE, **G**, **H** 4D flow and 2D flow, and **I**, **J** 4D flow MIP and 4D flow

**Fig. 5:**
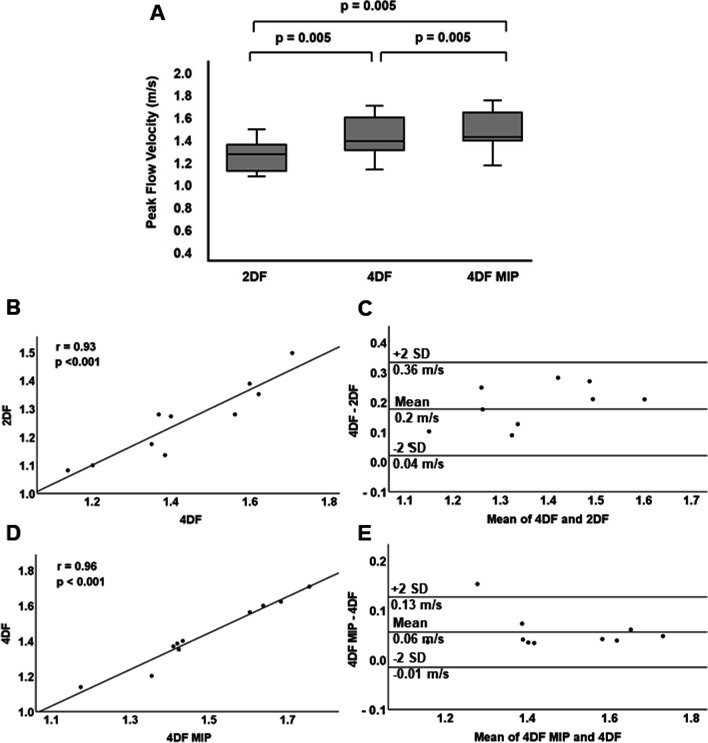
Peak flow velocity analyses of the healthy controls (n = 10). **A** Peak systolic flow measured with 2D flow, 4D flow, and 4D flow MIP. **B**–**E** Linear regression, Spearman’s correlation and Bland–Altman analyses of 4D flow and 2D flow (**B** and **C**), and 4D flow MIP and 4D flow (**D** and **E**)

The median peak aortic flow velocity in the patient population was 4.3 m/s (range 2.7–6.1 m/s) measured by TTE, and 4.5 m/s (2.9–6.5 m/s) by 2D flow CMR (Fig. 3A). The minor difference between 2D flow and TTE were insignificant (bias 0.07 m/s, limits of agreement ± 1.5 m/s) (Fig. 4A, B). The median peak aortic 2D flow after angle correction was 4.6 m/s (2.9–7.2 m/s), which was significantly higher than in both TTE (bias 0.3 m/s, limits of agreement ± 2.1 m/s; p < 0.001) and 2D flow before angle correction (bias 0.2 m/s, limits of agreement ± 0.5 m/s; p < 0.001) (Fig. 4C, D). The median distance of the 2D flow measurement plane from the valve orifice at cardiac systolic phase was 8 mm (0–14 mm). The median jet angle (offset from the 2D flow measurement plane) was 12° (1.0°–40.0°) corresponding to a 2.2% (0.0–23.4%) peak flow velocity estimation error. The mean forward flow volume at the same level was 78 ml ± 27 ml (Additional file 1: Fig. S1). In the non-valvular disease control population, median peak flow velocity by 2D flow was 1.3 m/s (1.1–1.5 m/s) (Fig. 5).

### CMR 4D flow

The median peak systolic 4D flow velocity in the patient population measured by the commercial Medis software (4D flow) was 3.1 m/s (range 1.7–5.1 m/s) (Fig. 3A), which was significantly lower compared to TTE (bias − 1.1 m/s, limits of agreement ± 1.4 m/s; p < 0.001) and 2D flow (bias − 1.2 m/s, limits of agreement ± 1.6 m/s; p < 0.001), as well as to angle-corrected 2D flow (bias − 1.4 m/s, limits of agreement ± 1.6 m/s; p < 0.001) (Fig. 4E–H). The 4D flow peak flow velocity was significantly lower when measured from the same plane with 2D flow or from the anatomically selected planes (Fig. 3B). The mean forward flow volume obtained by 4D flow at the level of the detected peak jet (112 ml ± 38 ml) was higher than in 2D flow measurements (p < 0.001) (Additional file 1: Fig. S1).

The median peak systolic 4D flow velocity measured by the non-commercial method (4D flow MIP) yielded slightly higher results than by the commercial 4D flow method (median 3.3 m/s, range 2.1–6.2 m/s; bias 0.13 m/s, limits of agreement ± 0.36 m/s; Figs. 3, 4, I, J), but lower compared to the other methods (bias to 2D flow − 1.0 m/s, limits of agreement ± 1.5 m/s).

There was a good agreement between the observers, the mean difference being 0.07 m/s (SD ± 0.5 m/s) and intraclass correlation coefficient 0.86. Peak flow velocity values measured by CAAS MR 4D Flow software were similar to those obtained with Medis QFlow 4D software (bias − 0.1 m/s, limits of agreement ± 0.96 m/s; p = 0.68). The results were similar in bicuspid and tricuspid valves (Additional file 2: Fig. S2).

In the non-valvular control group, the median peak flow velocity was higher in 4D flow (1.4 m/s; 1.1–1.7 m/s) than in 2D flow (bias 0.2 m/s; limits of agreement ± 0.16 m/s) and higher in 4D flow MIP (1.4 m/s; 1.2–1.8 m/s) than in 4D flow (bias 0.1; limits of agreement ± 0.07 m/s), the differences being statistically significant (Fig. 5).

## Discussion

In this prospective study, we examined 90 patients with severe symptomatic AS, to our knowledge the largest cohort so far in this patient group. CMR 2D flow showed excellent concordance with the TTE data, even when flow jets were mildly angulated compared to the measurement plane. Therefore, 2D flow constitutes a potential tool for AS evaluation when supplementary information is required due to e.g., poor visibility or discordant findings in TTE. Similar results have been reported in patients with moderate to severe AS using flow-derived aortic valve area calculation [11]. Our results are consistent with previous studies reporting optimal slice positioning at the level of the sinus of Valsalva, which is also favorable for the detection of possible regurgitation [11, 25]. In addition to flow assessment, CMR may provide useful diagnostic information by revealing myocardial scarring or other underlying myocardial conditions especially in patients with left ventricular hypertrophy [26].

We also present a method for correcting 2D peak flow velocity measurements for the angle between the skewed aortic velocity jet and the flow velocity measurement plane. The angle-corrected variable showed slightly more statistical dispersion than the original 2D flow measurements or TTE, rendering it possible that the angle measurement itself or the correction method contain some sources of error. Instead of an absolute means for correction, this method could rather be utilized in evaluating the magnitude of possible measurement error in 2D flow, taking possible underestimation of oblique jets into account in the final report.

In contrast to the single imaging plane in 2D flow CMR, the phase-contrast images in 4D flow are acquired in three dimensions, enabling the retrospective adjustment of the measuring angle and plane. The consequent more elaborate characterization of the flow features, in turn, is expected to result in more precise velocity values. Indeed, 4D flow has been shown to calculate peak velocity more accurately than 2D flow in non-stenotic bicuspid aortic valves of pediatric patients [23]. In a recent study, 4D flow-derived pressure gradients also showed good concordance with invasive assessment [27]. Indeed, 4D flow detected higher peak flow velocity values as compared to 2D flow in our control population with normal aortic flow range. At present, the wider availability of 2D flow and 4D flow being currently more time-consuming favor the use of 2D flow in daily practice. In our material, the 4D flow acquisition took more than 10 min per patient, whereas 2D flow throughplane images are obtained within a single breath-hold.

Interestingly, while CMR 4D flow performed well in the control population, it significantly underestimated peak flow velocity values compared with 2D flow and TTE in our selected patient material consisting of patients with severe AS. A similar tendency has been reported in a study describing otherwise superior properties, but lower flow-derived peak pressure gradients in 4D flow compared with TTE [12]. In contrast, flow volumes were higher in 4D flow than in 2D flow measurements, suggesting that the underestimation derives from the accelerated flow jet itself. All the 4D flow analysis methods used in this study underestimated peak flow velocities by approximately 1 m/s as compared to 2D flow and TTE, the MIP-based approach described by Rose et al. yielding slightly higher values than the commercially available methods.

Narrow and oblique jets are a known challenge in optimising CMR phase-contrast flow velocity measurements [28]. Indeed, a possible cause for the observed underestimation of the peak flow velocity is the intravoxel phase averaging due to a decreased spatial resolution in the 4D flow technique. Such limited spatial resolution is, however, needed to be able to reach sufficient signal level within a reasonable scan time. In the very narrow peak jets formed by the stenotic aortic valve orifice, even minor averaging may result in significant downgrading of the actual turbulent peak jet, and subsequent underestimation of peak aortic jet velocity. Such averaging phenomenon has been observed in pulmonary arteries, where considerable spread in the peak velocity values has been obtained by CMR 4D flow [29]. An interesting patient group that potentially benefits from multimodality imaging is low flow—low gradient AS [30]. Due to the low number of these patients in our material (n = 4), statistical analyses were not possible. The valve orifice and flow jet are, however, narrow also in this subgroup of AS patients. Therefore the decreased spatial resolution of 4D flow may result in a similar averaging phenomenon regardless of the lower peak flow velocity in low flow—low gradient aortic stenosis.

In most previous investigations, 4D flow has been described as a reliable and accurate technique for measuring aortic peak systolic flow velocity [13, 23, 31], and even suggested to be a superior method for the quantification of aortic peak velocity and the derived pressure gradients in assessing disease severity [7]. In contrast to some of these studies, our results indicate that 4D flow may not be as reliable in severe AS as in the milder stages of the disease [7]. In some study designs, the severity of AS has been determined based on the 4D flow data [32]. The possible underestimation of aortic peak flow velocity in severe AS may, however, result in a systematic error in this particular patient characterization, categorizing some of the severe AS as moderate. Therefore, confirming 4D peak flow velocity measurements with another method may currently be advisable, especially when evaluating the patient’s need for intervention. In fact, an alternative method using effective valve orifice calculation from 4D flow independently of peak velocity measurements has already been described [7].

### Study limitations

The limited temporal resolution of CMR flow data may constitute a source of error in this study. Our 4D flow settings are, however, a compromise between a reasonable scan time and spatial resolution, and solutions leading to a prolonged scan length may restrict the clinical usability of this technique in some populations. The acquisition voxel size may also affect the peak flow velocity bias. Thus, decreasing the voxel size could have resulted in lower bias values. Decreasing the voxel size, would, however, also increase the image noise. Therefore, more averaging would have been required to reach an acceptable signal-to-noise ratio, ultimately leading to heavily prolonged scan length.

## Conclusions

2D flow CMR may be used to measure aortic peak systolic flow velocity in a routine clinical workflow at 1.5T even in severe cases of AS. 2D flow may therefore provide additional information in the evaluation of AS severity and need for interventions. Further research is needed to verify and hopefully overcome the issues with measuring peak flow velocity by 4D flow in severe AS, which may derive from the intravoxel averaging of the narrow peak jets.

## Supplementary Information

**Additional file 1: Fig. S1.** CMR left ventricular stroke volume and forward flow volume by 2D flow and 4D flow (n = 90).

**Additional file 2: Fig. S2.** 2D flow and 4D flow peak flow velocity in bicuspid and tricuspid valves (total n = 90).

## Data Availability

The datasets analysed during the current study are not publicly available due to planned future publications.

## Abbreviations

2D: Two-dimensional
4Ch: Four-chamber
4D: Four-dimensional
AS: Aortic valve stenosis
AVA: Aortic valve area
bSSFP: Balanced steady state free precession
CMR: Cardiovascular magnetic resonance
ECG: Electrocardiogram
FOV: Field-of-view
GRAPPA: Generalized autocalibrating partially parallel acquisition
LVEDV: Left ventricular end-diastolic volume
LVEF: Left ventricular ejection fraction
LVESV: Left ventricular end-systolic volume
LVOT: Left ventricular outflow tract
MIP: Maximum intensity projection
TAVI: Transcatheter aortic valve implantation
TE: Time to echo
TR: Repetition time
TTE: Transthoracic echocardiography
VENC: Velocity encoded
