# Supplementary figures and images for: Peak flow measurements in patients with severe aortic stenosis: a prospective comparative study between cardiovascular magnetic resonance 2D and 4D flow and transthoracic echocardiography
Source: J Cardiovasc Magn Reson. 2021 Nov 15;23:132. doi: 10.1186/s12968-021-00825-1 (PMC8591846; doi:10.1186/s12968-021-00825-1)

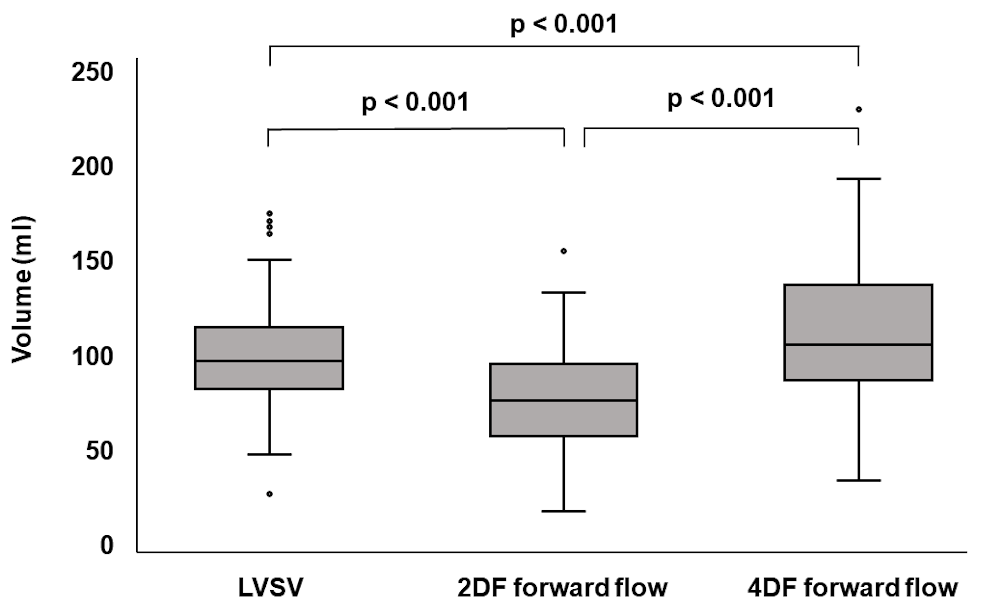

Supplement: Supplementary file 1 — Additional file 1: Fig. S1. CMR left ventricular stroke volume and forward flow volume by 2D flow and 4D flow (n = 90). [file 12968_2021_825_MOESM1_ESM.tif]

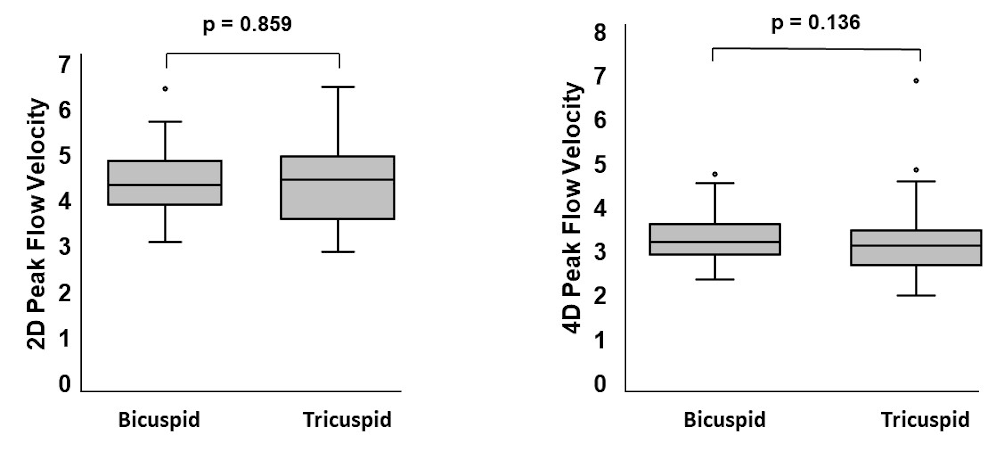

Supplement: Supplementary file 2 — Additional file 2: Fig. S2. 2D flow and 4D flow peak flow velocity in bicuspid and tricuspid valves (total n = 90). [file 12968_2021_825_MOESM2_ESM.tif]
